# Type 1 FSHD with 6–10 Repeated Units: Factors Underlying Severity in Index Cases and Disease Penetrance in Their Relatives Attention

**DOI:** 10.3390/ijms21062221

**Published:** 2020-03-23

**Authors:** Emmanuelle Salort-Campana, Farzad Fatehi, Sadia Beloribi-Djefaflia, Stéphane Roche, Karine Nguyen, Rafaelle Bernard, Pascal Cintas, Guilhem Solé, Françoise Bouhour, Elisabeth Ollagnon, Sabrina Sacconi, Andoni Echaniz-Laguna, Thierry Kuntzer, Nicolas Levy, Frédérique Magdinier, Shahram Attarian

**Affiliations:** 1 Reference Center of Neuromuscular disorders and ALS, Timone University Hospital, AP-HM, 264 rue Saint-Pierre, Cedex 05 13385 Marseille, France; emmanuelle.salort-campana@ap-hm.fr (E.S.-C.); fatehifa@gmail.com (F.F.); sadia.beloribi@ap-hm.fr (S.B.-D.); 2 Medical Genetics, Aix Marseille Université—Inserm UMR_1251, 13005 Marseille, France; stephane.roche@univ-amu.fr (S.R.); karine.nguyen@ap-hm.fr (K.N.); rafaelle.bernard@ap-hm.fr (R.B.); nicolas.levy@univ-amu.fr (N.L.); Frederique.MAGDINIER@univ-amu.fr (F.M.); 3 Service de Neurologie et d’explorations fonctionnelles, Centre Hospitalier Universitaire de Toulouse, 31000 Toulouse, France; cintas.p@chu-toulouse.fr; 4 Reference Center of Neuromuscular Disorders AOC, Bordeaux University Hospitals, 33000 Bordeaux, France; guilhem.sole@chu-bordeaux.fr; 5 Electroneuromyography and Neuromuscular Department, GHE Neurologic Hospital, Cedex 69677 Lyon-Bron, France; francoise.bouhour@chu-lyon.fr; 6 Neurogenetic Department, GHN Croix-Rousse Hospital, 69004 Lyon, France; elisabeth.ollagnon@chu-lyon.fr; 7 Neuromuscular Disease Specialized Center, Nice University Hospital, 06000 Nice, France; sacconi.s@chu-nice.fr; 8 Neurology Department, APHP, CHU de Bicêtre, 78 rue du Général Leclerc, Cedex 94276 Le Kremlin-Bicêtre, France; andoni.echaniz-laguna@aphp.fr; 9 Nerve-Muscle Unit, Department of Clinical Neurosciences, Lausanne University, Hospital (CHUV), Lausanne 1002, Switzerland; thierry.kuntzer@chuv.ch

**Keywords:** Facioscapulohumeral muscular dystrophy, FSHD, phenotype, genotype, association, correlation, methylation

## Abstract

Molecular defects in type 1 facioscapulohumeral muscular dystrophy (FSHD) are caused by a heterozygous contraction of the D4Z4 repeat array from 1 to 10 repeat units (RUs) on 4q35. This study compared (1) the phenotype and severity of FSHD1 between patients carrying 6–8 vs. 9–10 RUs, (2) the amount of methylation in different D4Z4 regions between patients with FSHD1 with different clinical severity scores (CSS). This cross-sectional multicenter study was conducted to measure functional scales and for genetic analysis. Patients were classified into two categories according to RUs: Group 1, 6–8; Group 2, 9–10. Methylation analysis was performed in 27 patients. A total of 99 carriers of a contracted D4Z4 array were examined. No significant correlations between RUs and CSS (r = 0.04, *p* = 0.73) and any of the clinical outcome scales were observed between the two groups. Hypomethylation was significantly more pronounced in patients with high CSS (>3.5) than those with low CSS (<1.5) (in DR1 and 5P), indicating that the extent of hypomethylation might modulate disease severity. In Group 1, the disease severity is not strongly correlated with the allele size and is mostly correlated with the methylation of D4Z4 regions.

## 1. Introduction

Facioscapulohumeral muscular dystrophy (FSHD1; OMIM 158900) is a common form of muscular dystrophy, affecting 1 in 20,000 to 1 in 8000 people, characterized by asymmetric and progressive weakness of the facial, shoulder girdle, and upper arm muscles [1,2,3,4], but often also with subsequent lower limb involvement. FSHD is an autosomal dominant disorder with variable severity, inter- and intra-familial heterogeneity [5], and incomplete penetrance. Its molecular genetic basis is highly complex [6]. The disease locus was mapped to the subtelomeric region of the long arm of chromosome 4q35, according to the genetic linkage analysis [7]. The molecular defect of type 1 FSHD (FSHD1) results from a heterozygous contraction of the D4Z4 repeat array from 1 to 10 repeat units (RUs) on 4q35. D4Z4 contraction is considered pathogenic if it occurs on a specific chromosomal background, i.e., (i) the presence of a 4qA haplotype and (ii) a single nucleotide polymorphism that creates a polyadenylation site for the distal DUX4 transcript [8,9,10]. The pathological cut-off is conventionally determined at 10 RUs, and the majority of patients with FSHD1 carry 1–8 RUs on one allele [11]. Large studies provided data on the expressivity and penetrance in the 1–6 RU range; however, the size of the D4Z4 allele could not predict the severity of clinical outcomes at all times [12,13,14], and only a few studies reported the phenotypic spectrum associated with larger pathological alleles (>6 RUs) [11]. In FSHD1, the reduced size of the D4Z4 array is associated with hypomethylation of the repetitive element, whereas in FSHD2, decreased DNA methylation is more pronounced and often segregates with SMCHD1 gene mutation on chromosome 18p [15]. Changes in this epigenetic mark have been associated with the clinical expression of FSHD1 [1,16], and in a subset of patients carrying borderline D4Z4 arrays and SMCHD1 mutations [17].

Our previous study [18] has shown that the penetrance was lower in patients with 9–10 RUs than in those with 6–8 RUs. Here, we aimed at comparing the phenotype and severity between patients with FSHD1 carrying 6–8 RUs and those carrying 9–10 RUs. In addition, the level of methylation in different D4Z4 regions was compared between patients with FSHD1 with different clinical severities to identify the main factor (i.e., RUs or methylation) associated with the disease severity in patients with 6–10 RUs. Through a multivariate analysis and a binary logistic regression, we also tried to identify how several factors synergistically influence CSS.

## 2. Results

### 2.1. Patient Demographic Characteristics

A total of 99 carriers of a contracted D4Z4 array (65 index cases and 34 relatives) from 65 unrelated families were examined, and 62 (62.6%) of them were men. The number of patients (ICs) in Group 1 (6–8 RUs) was 49 (75.4%) and in Group 2 (9–10 RUs), 16 (24.6%). The mean ± standard deviation age of patients was 51 ± 15.8 (Group 1, 51.2 ± 14.2; Group 2, 51.3 ± 17.6; *p* = 0.34) years. The number of women in Group 1 (ICs) was 14 (28.6 %), and in Group 2, 7 (43.8 %).

### 2.2. Age of Onset, RUs, and CSS

In index cases, the mean age at diagnosis was 44.2 ± 15.9 (Group 1, 44.1 ± 15.4; Group 2, 44.6 ± 18.1; *p* = 0.94) years. The mean age at the onset of upper limb weakness was 33.4 ± 17.0 (Group 1, 34.0 ± 17.6; Group 2, 34.0 ± 15.8; *p* = 0.95) years and that of the lower limbs was 41.4 ± 15.7 (Group 1, 41.7 ± 15.8; Group 2, 40.1 ± 16.2; *p* = 0.96) years. RUs were not significantly correlated with CSS (r = 0.17, *p* = 0.51) as well as adjusted mini motor test sum (SMMT) (r = 0.04, *p* = 0.71).

### 2.3. Comparison of Clinical Data Between the Two Groups

Results of the comparison between the two groups according to RUs are summarized in Table 1. No significant difference was observed in terms of any clinical evaluations between the two groups including adjusted SMMT, CSS, Brooke, Gardner, Brooke/Gardner sum-score, MFM score, Time to Walk 10 m, Time to climb 4 steps, Time of Barré (s), Time of Mingazzini (s), ABDUR, ABDUL, ANTER, and ANTEL (Table 1).

In addition, no significantly different frequency of abdominal weakness (χ² = 0.98, *p* = 0.32), facial involvement (χ² = 1.28, *p* =0.26), asymmetrical weakness (χ² = 0.18, *p* = 0.67), and steppage gait (χ² = 0.43, *p* = 0.51) was observed between the two groups. Remarkably, seven patients (10.8%) were wheelchair users with a higher proportion of patients in Group 1 vs. Group 2 (6/1), but the difference did not reach significance.

### 2.4. Association Between FSHD Severity DNA Methylation

DNA methylation has been proposed as a potential modifier of FSHD severity. To test this association in our cohort, we analyzed D4Z4 methylation profile in an equivalent number of randomly selected severely affected patients on the one hand (clinical score, 3.5–5 in 13 samples), and patients who are mildly affected on the other hand (clinical score, 0.5–1.5 in 14 samples), i.e. 27 patients among the 99 cases included in the study.

**Figure 1:**
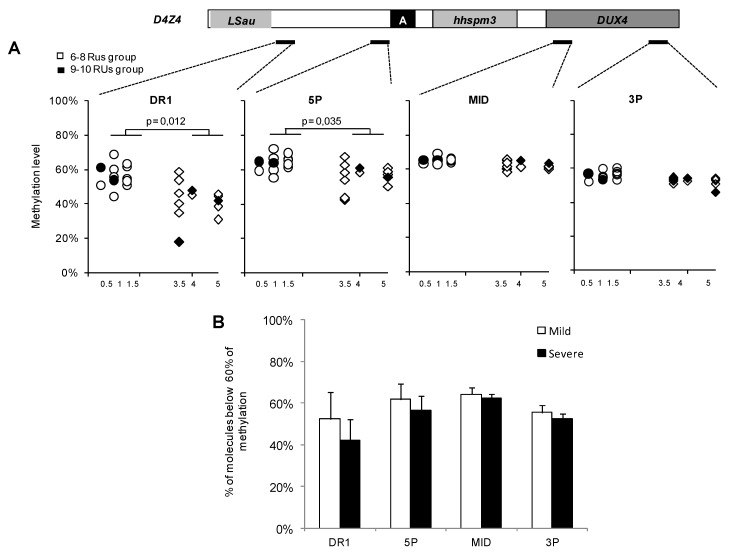
The percentage of methylation in patients with a low severity score (<1.5) compared to patients with a high severity (>3.5), patients from the 6–8 repeat units (RUs) group are represented by white dots, patients from the 9–10 RUs group are represented by black dots (panel **A**). The percentage of DNA molecules below 60% of the methylation level is also illustrated (panel **B**). Hypomethylation was more significantly pronounced in patients with a high severity score than in those with a low severity score, especially for the proximal D4Z4 region (DR1 and 5P).

Figure 1 displays the percentage of methylation in patients with a low severity score (<1.5) compared to patients with a high severity score (>3.5) (panel A). DNA methylation was assessed for four regions across D4Z4, the DR1, and 5P sequences, which are differentially methylated in the disease and the MID and 3P sequences for which stable methylation has been reported [19]. We also report the percentage of DNA molecules with low methylation levels (i.e., the percentage of molecules showing a level of methylation <60%, panel B). By analyzing the global percentage of methylation in the two categories, we observed that hypomethylation was more significantly pronounced in patients with a high severity score than in those with a low severity score for the proximal D4Z4 region (DR1 and 5P), suggesting that in these two groups, hypomethylation might be associated with disease severity.

We then calculated the correlation between CSS and methylation level. Although on a small subgroup of patients, significant correlations were found with different parameters. The correlation coefficient between CSS and global methylation level (%) for different D4Z4 regions was significant: DR1: r = −0.47, *p* = 0.01; 5P: r = −0.42, *p* = 0.03; MID: r = −0.51, *p* = 0.01; 3P: r = −0.43, r = 0.03 (Table 2). The correlation between CSS and the percentage of hypomethylated molecules was solely significant in the DR1 region: r = 0.42, *p* = 0.03 (Table 2).

To test whether functional parameters used for patients assessment might be associated with methylation changes, we calculated the correlation between DNA methylation and functional scores of Brooke, Gardner, and time to walk 10 min, time to climb 4 steps, time of Barré, time of Mingazzini, ABDUR, ABDUL, ANTER, and ANTEL (Table 2). The time of Barré was significantly positively correlated with the percentage of methylation in DR1, 5P, MID, and 3P. However, no significant correlation was observed between the amount of methylation and other scores (Table 2). Further investigations on a larger cohort of samples would be required to ascertain the link between DNA methylation, functional parameters, disease severity, and the number of residual repeats.

## 3. Discussion

The contraction of D4Z4 repeats at the 4q35 locus has been associated with FSHD1. Upon contraction, the size of the residual array has been considered as a determinant in the clinical phenotype, age of onset, and disease severity. However, given the variability of disease presentation among patients, incomplete penetrance, and familial heterogeneity, genotype–phenotype correlations remain a challenge for genetic counseling or prediction of the clinical disease progression.

In this cross-sectional multicenter study, the phenotype–genotype correlation was evaluated in 99 patients carrying contracted alleles of 6−10 RUs corresponding to 65 unrelated families. Our previous study [18] reported that penetrance in this range of alleles was low (57%) and incomplete. Furthermore, penetrance was found to be lower in patients with 9–10 RUs (47%) than in those with 6–8 RUs (62%), as described in other reports [11,12]. However, although penetrance inversely decreases with the number of RUs, the RU number does not predict the clinical severity in patients with 6–10 RUs. Based on our results, the phenotype–genotype correlation in patients with FSHD1 with higher RUs is not as obvious as in patients with lower RUs. Due to differences in disease severity, from the absence of clinical alterations to more severe clinical outcomes in patients with higher RUs, this range may be considered as a gray zone for the phenotype–genotype correlation, for which factors, other than the number of RUs, are involved in the disease severity.

*Genotype–phenotype correlation*. Regarding the D4Z4 repeat number, the correlation between clinical severity and RUs has been found, with individuals carrying 1–3 RUs typically representing the most severe end of the disease spectrum with earlier onset and more severe disease presentations [20,21,22,23]. However, differences in disease severity have been reported in patients carrying a short allele (1–3 RUs), ranging from very severe to milder forms or asymptomatic carriers [24,25]. Two recent studies have reported that the size of contracted alleles has no definitive prognostic value for disease severity [12,13], and a short 1–3 RUs allele was not always predictive of a severe clinical outcome with relatives carrying an allele of 1–3 RUs, exhibiting clinical variability from healthy subjects to patients with severe motor impairment [13]. At the other end of the spectrum, patients with contracted alleles of 7–10 RUs have been suggested to have a milder and later phenotype [11], with the possible exception of patients carrying a mutation in a modifier gene such as SMCHD [17], or patients inheriting an additional neuromuscular condition coincidentally [26,27]. 

In agreement, in a prospective cross-sectional observational study of 74 clinically affected patients with FSHD1, clinical severity measurements (measured by sum score MMT) between patients with 1–6 D4Z4 repeats and 7–10 repeats demonstrated that the residual repeat size in patients with 1–6 repeats had a linear effect on clinical severity, which was not found in those with 7–10 repeats [11]. However, thus far, no consistent correlation was found between fragment size and age at which ambulation was lost. [20]. Interestingly, in a Chinese cohort of 178 patients with 1–9 RUs, a significant typical inverse correlation was found between the EcoRI fragment size and clinical severity score [28], suggesting that differences might also depend on the size range considered for correlation analyses.

*FSHD severity and DNA methylation*. It recently became evident that the SMCHD1 epigenetic modifier has a role in FSHD [29], as well as in the regulation of D4Z4 methylation [30]. Although we did not evaluate methylation in all patients, hypomethylation was overall more significant in patients randomly selected in the two subgroups tested, i.e., patients with a high severity score (CSS >3.5) as compared to patients with a low severity score (CSS <1.5), particularly for the proximal D4Z4 region (DR1 and 5P). Significant correlations were found between the global amount of methylation and CSS, suggesting that the level of methylation might contribute to disease severity [31]. However, we found that RUs were not significantly associated with CSS, indicating that the FSHD severity is hardly predictable in patients with 6–10 RUs. DNA methylation was not tested in the group of patients with an intermediate score of severity, but by randomly selecting patients in the “low” and “high” severity score groups, disease severity was found to be correlated with DNA methylation.

Compared to healthy individuals or asymptomatic carriers, D4Z4 methylation is lower in patients with FSHD1 and more markedly decreased in patients with FSHD2. [16,31,32]. Due to the inclusion of both small and large families, our study has some limitations. There was a high proportion of index cases among affected carriers. We may suppose that index cases are more severe than carriers’ relatives. This is a bias for analysis of the severity.

The bivariate correlation analysis showed that all the potential CSS predictive factors are independent. Despite the fact that the full model containing all predictors (four percentage of methylation variables) was significant (chi2 = 5.53, *p* = 0.02), the only percentage of methylation of the 3P region could make a statistically significant contribution to the model recording an odds ratio of 0.71 (*p* = 0.049).

In conclusion, our study indicates that the disease severity is not strongly correlated with the size of the allele (RUs) in the upper range of RUs in FSHD1 (6–10 RUs), but that the level of methylation might be more variable in severely affected patients compared to mildly affected cases. However, these results have to be taken with caution, and even if a global trend is observed when comparing the two groups of patients, the use of DNA methylation as a prognostic marker at the individual level remains difficult. This further highlights the need of a complete understanding on the role and regulation of this epigenetic modification in the disease.

## 4. Materials and Methods

### 4.1. Patients

A cross-sectional multicenter study was conducted in 6 French and 1 Swiss neuromuscular center (for more information, see Campana-Salort et al. Orphanet J Rare Dis 2015) [18]. Among 65 FSHD1 families, examined in the reference centers from 2007 to 2009, 184 subjects were included in the study. All patients had a clinical examination by expert neurologists. All index cases (IC) harbored a 4qA contracted allele. Among the 119 relatives, 59 were carriers of the D4Z4 contraction identified in the IC, and 60 were non-carriers. Among the 59 carriers’ relatives, based on clinical examination by expert neurologists, 34 were defined as clinically affected carriers, and 25 were unaffected carriers. Individuals carrying a contracted D4Z4 array with an estimated size of 6 RUs (27kb) to 10 RUs (40 kb) (index cases; IC, *n*= 65) and their clinically affected carriers’ relatives (*n* = 34) were selected. For ethical considerations, patients younger than 18 years of age or pregnant women were not included.

All patients enrolled provided informed consent to participate in the study. This study was approved by the local ethics committee (Comité de Protection des Personnes Sud Mediterranée I).

### 4.2. Clinical and Functional Evaluation

Neurologists performed clinical examination of all relatives blinded to their genetic results, and the status (clinical affected or asymptomatic carriers) were defined based on the clinical examination. For each patient, the following data were recorded: Medical history, pedigree, clinical examination, functional assessment, and manual muscular test results. The first symptom experienced and the age of onset was recorded for ICs only. 

Neurologists examined the presence or absence of scapular winging, facial, limb, and thoracoabdominal muscle weakness, selective involvement, and asymmetry of muscle weakness. The functional evaluation included manual muscle testing (MMT, according to Medical Research Council), clinical severity scale (CSS) [20]. Motor Function Measure scale (MFM) [33], and functional grades of Brooke and Vignos scales for the upper limb [34], and Gardner-Medwin Walton (Gardner) for the lower limbs [35]. Timed motor tests were also performed including the time to walk 10 min, climb 4 steps, tests of time of Barré (position of arms stretched forward to be maintained), time of Mingazzini (supine, legs flexed), degree of upper limb abduction and anterior flexion (right upper limb abduction [ABDUR], left upper limb abduction [ABDUL], right upper limb anterior flexion [ANTER], and left upper limb anterior flexion [ANTEL]). The shoulder range of motion was categorized into 4 grades: 1, 0–45°; 2, 46–90°; 3, 91–135°; and 4, 136–180°.

Sum-score MMT (SMMT) was used to measure the strength in 36 muscle groups according to the MRC scale (graded from 0 to 5) and 4 muscle groups of the face (graded from 0 to 3). Normal SMMT is 242, the total scores of 40 muscles examined.

To adjust for the difference in age among patients, the SMMT score was divided according to patients’ age to obtain an overall rate: Adjusted SMMT= (242-SMMT) × 100/age (lower values of adjusted score corresponding to a better test result).

### 4.3. Genetic Testing/Methylation Profile Analysis

All genetic analyses were performed at the Laboratory of Molecular Genetics in the Department of Medical Genetics in Marseille by Molecular Combing or Southern blotting, as previously described [18]. Patients were arbitrarily divided into 2 categories according to RUs: Group 1, 6–8 RUs, and Group 2, 9–10 RUs.

To determine whether clinical severity was associated with DNA methylation changes, this epigenetic mark was analyzed in the 2 subgroups: Severely affected (CSS between 3.5 and 5) and mildly affected (CSS between 0.5 and 1.5) using the blood DNA after the chemical modification with sodium bisulfite and deep sequencing for a total of 27 patients randomly selected out of the 99 included in the study. For each sample, 4 different regions within D4Z4 were analyzed [16,36]. For each region, thousands of DNA molecules were sequenced on average, with global coverage of 82%. 

The level of methylation was calculated as the mean methylation level for all CpG in a given sequence for all DNA molecules analyzed, as described in Roche et al. [19]. All 4q alleles were analyzed for 4 different regions across D4Z4 (DR1, 5P, Mid, and 3P) with 3 two different regions, which were invariable in the different context (healthy individuals, affected patients, MID and 3P) and for which DNA methylation was not subjected to changes depending on the number of copies as described in Roche et al. [19]

### 4.4. Statistical Analyses

For data analyses, the R Studio software (R version 3.1.2) was used. Shapiro–Wilk was used to determine the normal distribution of data. Since clinical measurements did not follow a normal distribution, the Mann–Whitney test was used to compare the clinical outcome measures between the two groups (6–8 vs. 9–10 RUs). To compare the categorical data (i.e., abdominal weakness, facial involvement, asymmetrical weakness, and steppage gait), the chi-square test (χ² test) was used. In addition, the correlation between RUs and age of onset in the upper and lower limbs, as well as the correlation between methylation values and clinical scores, was measured using the Spearman correlation analysis. To compare the percentage of methylation in patients with a low severity score (CSS <1.5) to patients with a high severity (CSS >3.5), the Mann–Whitney test was used. Data were reported as median with a confidence interval (25–75 percentile). Differences were considered significant for all analyses when the *p*-values were <0.05. To assess the influence of the different factors on the disease severity, we performed a binary logistic regression, as the CSS does not follow the abnormal distribution. We conducted a bivariate correlation analysis (Spearman correlation analysis) between CSS and factors such as age at the onset, sex, RUs, and D4Z4 methylations, and we considered factors with correlation significance < 0.1 in the logistic regression equation.

## Figures and Tables

**Table 1:** Comparison of clinical scales between the two groups of patients with facioscapulohumeral muscular dystrophy (FSHD) (6–8 RUs vs. 9–10 RUs).

	6–8 RUs (*n* = 74)	9–10 RUs (*n* = 25)	Z	*p*
Adjusted SMMT	0.67 (0.32–1.07)	0.52 (0.28–0.99)	−0.38	0.70
CSS	2.5 (1.4–3.5)	2.5 (1.0–3.3)	−0.51	0.61
Brooke	2 (1–3)	2 (1–2.75)	−0.09	0.93
Gardner	2 (0–3)	1 (0–2)	−1.37	0.17
Brooke/Gardner sum-score	4 (2–5)	3 (2–4)	−1.2	0.23
MFM score	92.5 (81.5–99)	95.5 (86.25–98)	−0.56	0.58
Time to Walk 10 m	7 (6–11)	8 (6–9.75)	−0.34	0.74
Time to climb 4 steps	3 (2–5)	3.5 (2–7)	−023	0.82
Time of Barré (s)	60 (30–143)	92 (30–150)	−99	0.32
Time of Mingazzini (s)	60 (26.25–124.75)	75 (60–132)	−1.6	0.12
ABDUR	90 (80–180)	120 (80–180)	−0.64	0.52
ABDUL	90 (80–180)	170 (75–180)	−0.41	0.68
ANTER	110 (88.75–180)	120 (75–180)	−0.12	0.92
ANTEL	110 (90–180)	170 (75–180)	−0.47	0.64

**Table 2:** The correlation between functional scales and methylation.

	Percentage of Methylation					Percentage of Hypomethylation			
		DR1	5P	Mid	3P	DR1	5P	Mid	3P
**SMMT**	r	−0.31	−0.26	−0.35	−0.33	0.27	0.31	0.21	0.30
	*p*	0.12	0.12	0.08	0.10	0.18	0.12	0.30	0.14
**CSS**	r	−0.47	−.042	−0.51	−0.43	0.42	0.31	0.23	0.29
	*p*	0.01	0.03	0.01	0.03	0.03	0.12	0.26	0.14
**Brooke**	r	−0.22	−0.15	−0.21	−0.12	0.15	0.09	−0.06	0
	*p*	0.26	0.45	0.29	0.55	0.45	0.65	0.78	0.99
**Gardner**	r	−0.37	−0.29	−0.37	−0.34	0.33	0.18	0.17	0.2
	*p*	0.06	0.15	0.06	0.08	0.09	0.38	0.39	0.31
**Time to Walk 10 m**	r	−0.04	0.1	−0.16	−0.1	−0.07	−0.08	0.17	0.09
	*p*	0.86	0.63	0.42	0.63	0.74	0.7	0.4	0.67
**Time to climb 4 steps**	r	−0.04	0.08	−0.03	−0.02	−0.08	0	0.3	0.23
	*p*	0.86	0.71	0.89	0.93	0.72	0.99	0.15	0.27
**Time of Barré**	r	0.48	0.46	0.5	0.39	−0.52	−0.08	−0.33	−0.01
	*p*	0.01	0.02	0.01	0.05	0.01	0.7	0.1	0.95
**Time of Mingazzini**	r	0.19	0.17	0.17	0.17	−0.22	−0.26	−0.22	−0.07
	*p*	0.39	0.44	0.44	0.44	0.31	0.23	0.32	0.74
**ABDUR**	r	0.01	0	0.05	0.13	0.04	0.03	0.15	0.02
	*p*	0.95	0.99	0.8	0.54	0.86	0.89	0.48	0.92
**ABDUL**	r	0.06	0.08	0.06	0.24	−0.09	−0.14	−0.04	−0.28
	*p*	0.78	0.7	0.75	0.25	0.66	0.48	0.86	0.16
**ANTER**	r	0.12	0.09	0.15	0.3	−0.06	−0.01	0	−0.19
	*p*	0.57	0.66	0.47	0.14	0.76	0.97	0.99	0.36
**ANTEL**	r	0.14	0.13	0.12	0.34	−0.14	−0.16	−0.18	−0.47
	*p*	0.51	0.54	0.54	0.09	0.48	0.44	0.38	0.02

SMMT: Sum score MMT, CSS: Clinical severity score, ABDUR: Right upper limb abduction, ABDUL: Left upper limb abduction, ANTER: Right upper limb anterior flexion, ANTEL: Left upper limb anterior flexion. Red data: highlight the clinically significant data

## Abbreviations

ABDUL	Left Upper Limb Abduction
ABDUL	Right Upper Abduction
ANTEL	Left Upper Limb Anterior Flexion
ANTER	Right Upper Limb Flexion
CSS	Clinical Severity Score
FSHD	Muscular Facioscapulohumeral Dystrophy
IC	Index case
MFM	Motor Function Measure
RU	Repeat Unit
SMMT	Mini Motor Test Sum
